# Comprehensive analysis of gene expression profiles of annulus fibrosus subtypes and hub genes in intervertebral disc degeneration

**DOI:** 10.18632/aging.205653

**Published:** 2024-03-13

**Authors:** Xiaokun Zhao, Jian Zhang, Jiahao Liu, Jinghong Yuan, Tianlong Wu, Xigao Cheng

**Affiliations:** 1 Department of Orthopedics, The Second Affiliated Hospital of Nanchang University, Nanchang, Jiangxi 330006, P.R. China; 2 Jiangxi Key Laboratory of Intervertebral Disc Disease, Nanchang University, Nanchang, Jiangxi 330006, P.R. China; 3 Institute of Minimally Invasive Orthopedics, Nanchang University, Nanchang, Jiangxi 330006, P.R. China

**Keywords:** intervertebral disc degeneration, annulus fibrosus, molecular subtype, WGCNA, machine learning

## Abstract

Intervertebral disc degeneration (IVDD) has been considered a major cause of low back pain. Therefore, further molecular subtypes of IVDD and identification of potential critical genes are urgently needed. First, consensus clustering was used to classify patients with IVDD into two subtypes and key module genes for subtyping were identified using weighted gene co-expression network analysis (WGCNA). Then, key module genes for the disease were identified by WGCNA. Subsequently, SVM and GLM were used to identify hub genes. Based on the above genes, a nomogram was constructed to predict the subtypes of IVDD. Finally, we find that ROM1 is lowered in IVDD and is linked to various cancer prognoses. The present work offers innovative diagnostic and therapeutic biomarkers for molecular subtypes of IVDD.

## INTRODUCTION

Low back pain (LBP) is a global challenge that can cause serious health and socioeconomic burdens. Nearly 80% of people will suffer from this condition in their lifetime [1, 2]. The current etiology attributes the pain to intervertebral disc degeneration (IVDD) [3]. Ultimately, IVDD will irreversibly impair neurological function, adversely affecting both the individual and society [4]. At present, a multitude of therapy modalities have been suggested for the management of disc degeneration [5]. However, there is great heterogeneity in patients with disc degeneration, including clinical symptoms and postoperative prognosis. Hence, it is necessary to get a comprehensive comprehension of the distinct molecular subtypes associated with disc degeneration in order to enhance the prognosis of patients exhibiting varying subtypes of this condition.

Molecular biology bioinformatics has been used in clinical practice to screen pathways and biomarkers [6, 7]. Understanding the various situations of patients with IVDD is essential for clinicians to personalize treatment. However, few studies have explored molecular subtypes of IVDD. Wu et al. divided patients with IVDD into three subtypes based on 11 immune-related genes [8]. Nevertheless, the complete understanding of whether sequencing data from patients with IVDD can be used to identify specific molecular subtypes remains unclear.

The ferroptosis form of programmed cell death is characterized by phospholipid peroxidation and is controlled by intricate metabolic pathways within the cell [9]. These pathways include lipid metabolism, iron balance, redox homeostasis, and mitochondrial function. Ferroptosis is distinguished by phospholipid peroxidation. When it comes to the regulation of IVDD, the role that ferroptosis plays is a topic that is of great importance. Liu et al. used bioinformatics to reveal that iron ptosis occurs in IVDD, and that ferroptosis has the potential to enhance the improvement of IVDD by acting as an immune infiltrate [10]. According to Xiang et al., the results of ROC analysis and RT-qPCR validation indicate that most of the hub genes associated with ferroptosis have the ability to serve as hallmark genes for IVDD [11].

The intervertebral disc (IVD) is composed of the nucleus pulposus (NP), annulus fibrosus (AF), and cartilage endplates [12, 13]. As part of the rounded exterior of the IVD, the tough AF surrounds the soft interior NP. The most important function of the AF is to prevent protrusion of the NP from the IVD by hydraulically sealing the NP and evenly distributing any pressure and force exerted on the IVD [14]. The AF dysfunction results in protrusion of the NP from the IVD, which can lead to clinical symptoms such as LBP and nerve damage. Therefore, we conducted an analysis of the transcriptome data based on the AF.

**Figure 1:**
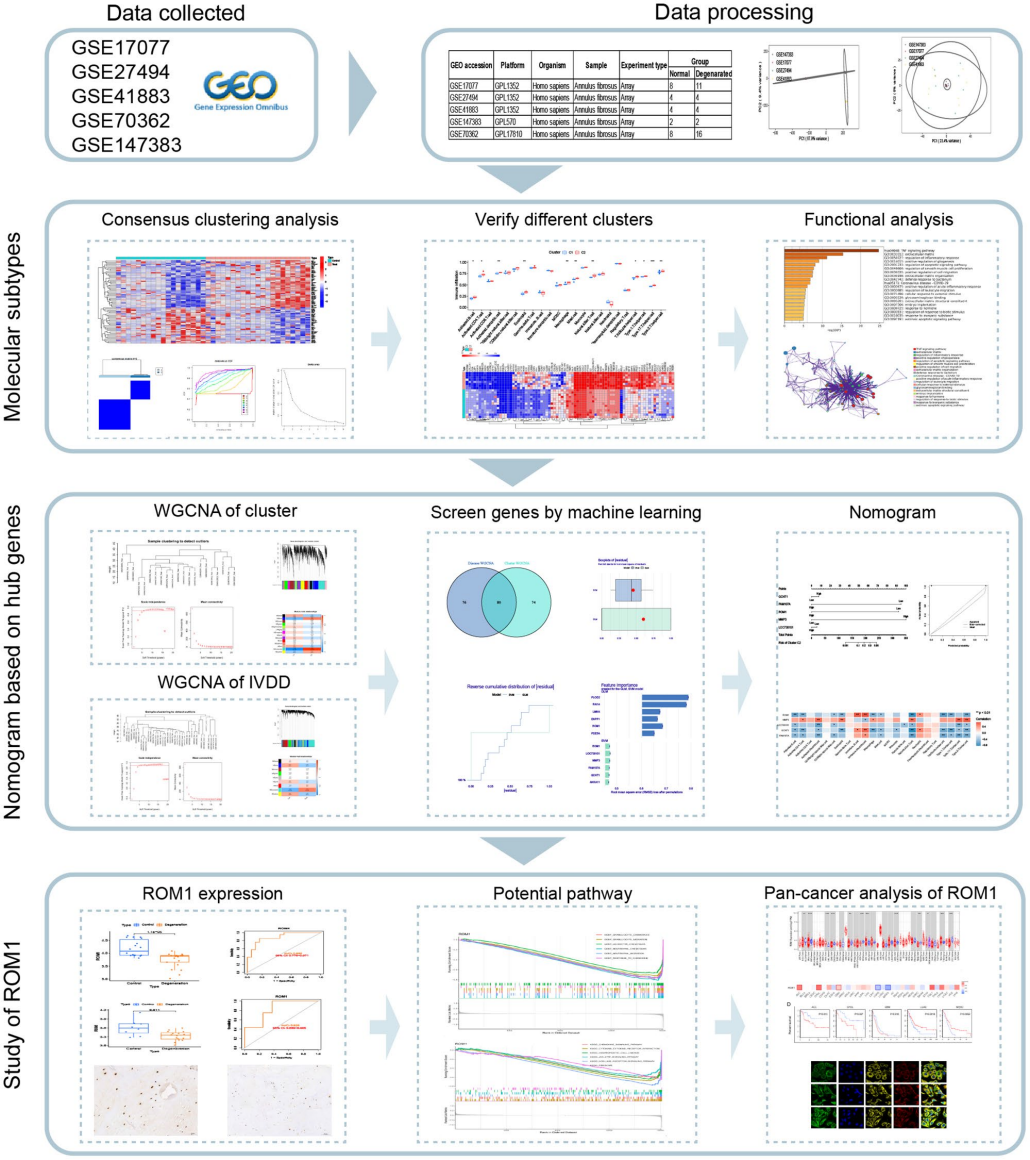
Research flow chart of the study.

In this study, the AF microarray dataset from the Gene Expression Omnibus (GEO) database was downloaded to identify differentially expressed genes (DEGs). Based on the differential expression of ferroptosis-related genes (DEFRGs), IVDD patients were classified into two subtypes by consensus clustering analysis. Then, a combination of weighted gene co-expression network analysis (WGCNA) and machine learning strategies was used for in-depth screening and identification of hub genes for IVDD. In addition, the correlation between hub genes and infiltrating immune cells was investigated to gain additional insights into the molecular immune mechanisms involved in the development of IVDD. Next, we verified low ROM1 expression in external datasets and clinical samples. Finally, single gene set enrichment analysis (GSEA) was used to reveal the underlying mechanism. The flow chart is shown in Figure 1.

## MATERIALS AND METHODS

### Data sources

The datasets used in the study were all obtained from the GEO database (https://www.ncbi.nlm.nih.gov/geo/). We downloaded the gene expression profiles from the GSE41883, GSE27494, GSE17077, GSE147383, and GSE70362 datasets. The GSE41883, GSE27494, GSE17077, and GSE147383 datasets were used as the training set, and GSE70362 was used as a validation set to verify biomarker diagnostic capabilities. The specific dataset information is shown in Table 1.

**Table 1:** Details of transcriptomic data.

**GEO accession**	**Platform**	**Organism**	**Sample**	**Experiment type**	**Group**	
					**Normal**	**Degenerated**
GSE17077	GPL1352	Homo sapiens	Annulus fibrosus	Array	8	11
GSE27494	GPL1352	Homo sapiens	Annulus fibrosus	Array	4	4
GSE41883	GPL1352	Homo sapiens	Annulus fibrosus	Array	4	4
GSE147383	GPL570	Homo sapiens	Annulus fibrosus	Array	2	2
GSE70362	GPL17810	Homo sapiens	Annulus fibrosus	Array	8	16

### Identification of DEFRGs

First, we combined the GSE41883, GSE27494, GSE17077, and GSE147383 datasets using the R package “SVA” and removed batch differences. Principal component analysis (PCA) plots were created using the R package “ggplot”. Ferroptosis-related genes (FRGs) were obtained from three databases, the specific sources of which were reported by Qu et al. [15]. We then extracted the expression matrices of FRGs. The R package “limma” was used to identify DEFRGs in the normal and degenerate groups, using *p* < 0.05 as the threshold for screening differential genes. The heatmap was displayed using the R package “pheatmap”.

### Consensus clustering based on DEFRGs

To investigate the role of DEFRGs in degenerating AF, we use the R package “ConsensusClusterPlus” to classify the degenerating AF into different subtypes based on DEFRGs. The grouping principles were as follows: (1) The cumulative distribution function (CDF) was small and grew slowly; (2) there were no small clusters or cross-clusters in the clustered data. Then, a PCA was performed to determine the success of the grouping. The Metascape online tool is a bioinformatics platform utilized for gene enrichment assessment [16] (http://metascape.org/).

### WGCNA analysis

WGCNA analysis was used to identify core genes for disease and typing separately, and then to identify the intersecting genes. First, sample outliers were filtered to make the model stable, and a suitable soft threshold β was selected and converted into a topological overlap matrix (TOM) and corresponding dissimilarity (1-TOM). The 1-TOM matrix was then used to construct a hierarchical clustering tree diagram to group genes with similar expression into different gene co-expression modules. The gene significance and module affiliation were calculated to assess the importance of gene and clinical information and to identify significant links between the modules and models.

### Screening of biomarkers by machine learning

First, we identified key genes by the intersection of the disease key module genes and the clustering key module genes. The “caret” package in R software was used to build the GLM and SVM models, using DEFRGs as the explanatory variable and patients with IVDD as the response variable. Then, the two models were analyzed using the “interpretation” function in the “DALEX” R package and the cumulative residual distribution was plotted to obtain the best model. Finally, we analyzed the importance of the variables in the prediction of the response variable. The ssGSEA analysis was conducted using the “GSVA” R package. The nomogram and calibration curve were plotted using the “rms” R package.

### Verification of ROM1

To test the validity of the biomarkers, the merged data set (comprising GSE147383, GSE17077, GSE274949, and GSE41883) was used as the training set and GSE70362 as the validation set. ROC curve-based studies were evaluated and the area under the curve (AUC) was calculated to assess the predictive effect achieved by the algorithm. *p* < 0.05 was considered statistically significant.

### IVD samples collection and immunohistochemical staining

A total of six recently obtained human disc tissue samples were procured from patients who were undergoing disc surgery at the Second Affiliated Hospital of Nanchang University. The Pfirrmann score was established by evaluating the preoperative MRI findings of each individual, and the samples were divided into normal and degenerative groups according to the Pfirrmann score. The researchers acquired informed consent from the patients who participated in this study. The application of clinical surgery was granted approval by the Medical Ethics Committee. The rabbit anti-ROM1 antibody was acquired from Proteintech (21984-1-AP).

### Single-gene GSEA

Single-gene GSEA was used to elucidate the underlying molecular mechanisms. We obtained “C2.cp.kegg.symbols” and “c5.go.symbols.gmt” gene set and used the “ClusterProfiler” R package for GSEA analysis [17]. GO enrichment analysis focuses on the description of biological processes (BP), cellular components (CC) and molecular functions (MF) associated with genes. The correlation coefficient of each gene with all genes in the gene set was ranked according to the expression value of each gene. The enrichment significance threshold was NOM *p*-value < 0.05 [18].

### Pan-cancer analysis of ROM1

The differential analysis of ROM1 between tumor tissues and their adjacent normal tissues was performed using the online website [19] (https://cistrome.shinyapps.io/timer/). The ROM1 prognostic effect on various tumors can be seen via the GEPIA [20] (http://gepia.cancer-pku.cn/). The measurement of ROM1 protein expression in diseased tissues was conducted by the Human Protein Atlas [21] (https://www.proteinatlas.org/), and verification of the protein’s localization at the subcellular level was accomplished. The protein-protein interaction (PPI) network obtained in this study was generated from the STRING database [22] (https://string-db.org/).

### Availability of data and materials

The datasets generated for this study can be found in the GEO database (GSE17077, GSE27494, GSE41883, GSE147383, and GSE70632; https://www.ncbi.nlm.nih.gov/geo/).

## RESULTS

### Consensus clustering analysis for IVDD

**Figure 2:**
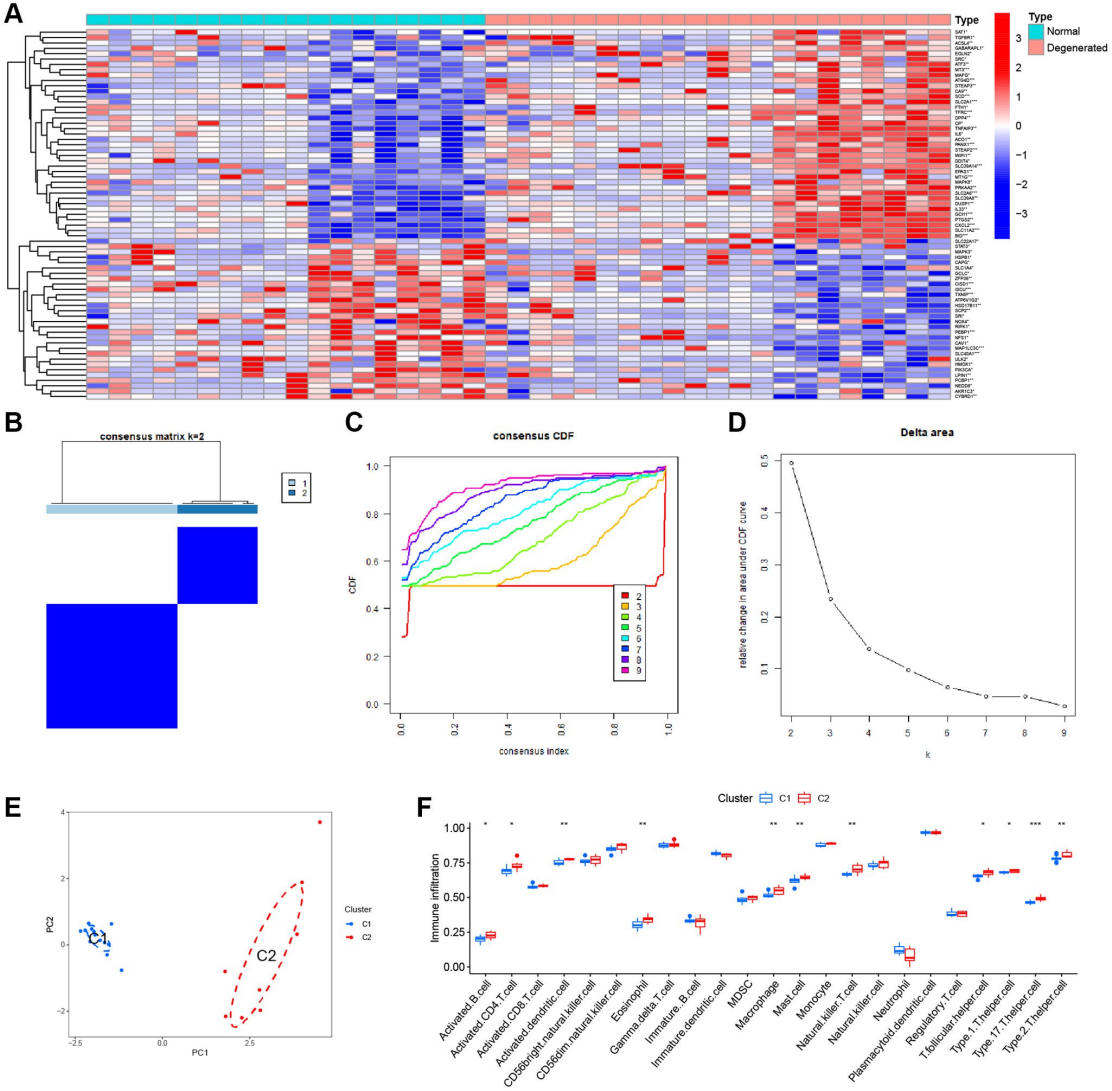
**Consensus clustering analysis.** (**A**) Heatmap of the DEFGs. (**B**) Cluster analysis. (**C**) Clustered consistent values and cumulative distribution functions (CDF) with different subgroup separations. (**D**) Relative changes in the area under the CDF curve at different subgroup spacing. (**E**) PCA of the two subtypes. (**F**) Differences in ssGSEA immune scores among different subtypes.

Four datasets were downloaded from the GEO database to compare gene expression levels between degenerated AF tissue and normal tissue. We performed batch correction using the R package “SVA”, and the results showed that the data were corrected at the same level (Supplementary Figure 1). There is an increasing number of studies indicating a potential association between ferroptosis and IVDD [23]. Given consideration of this, we performed a differential analysis on FRGs. The heatmap showed a total of 69 DEFRGs (Figure 2A). Based on the results of the above differential analysis, molecular cluster analysis was performed. The clustering results show that classification is most reliable and stable when k = 2 (C1: *n* = 13; C2: *n* = 8) (Figure 2B–2D). The PCA results showed high separation quality (Figure 2E). We found that cluster C2 had more immune cells, like active eosinophils, macrophage and mast cells, according to the ssGSEA results (Figure 2F).

### Functional analysis in different molecular subtypes

**Figure 3:**
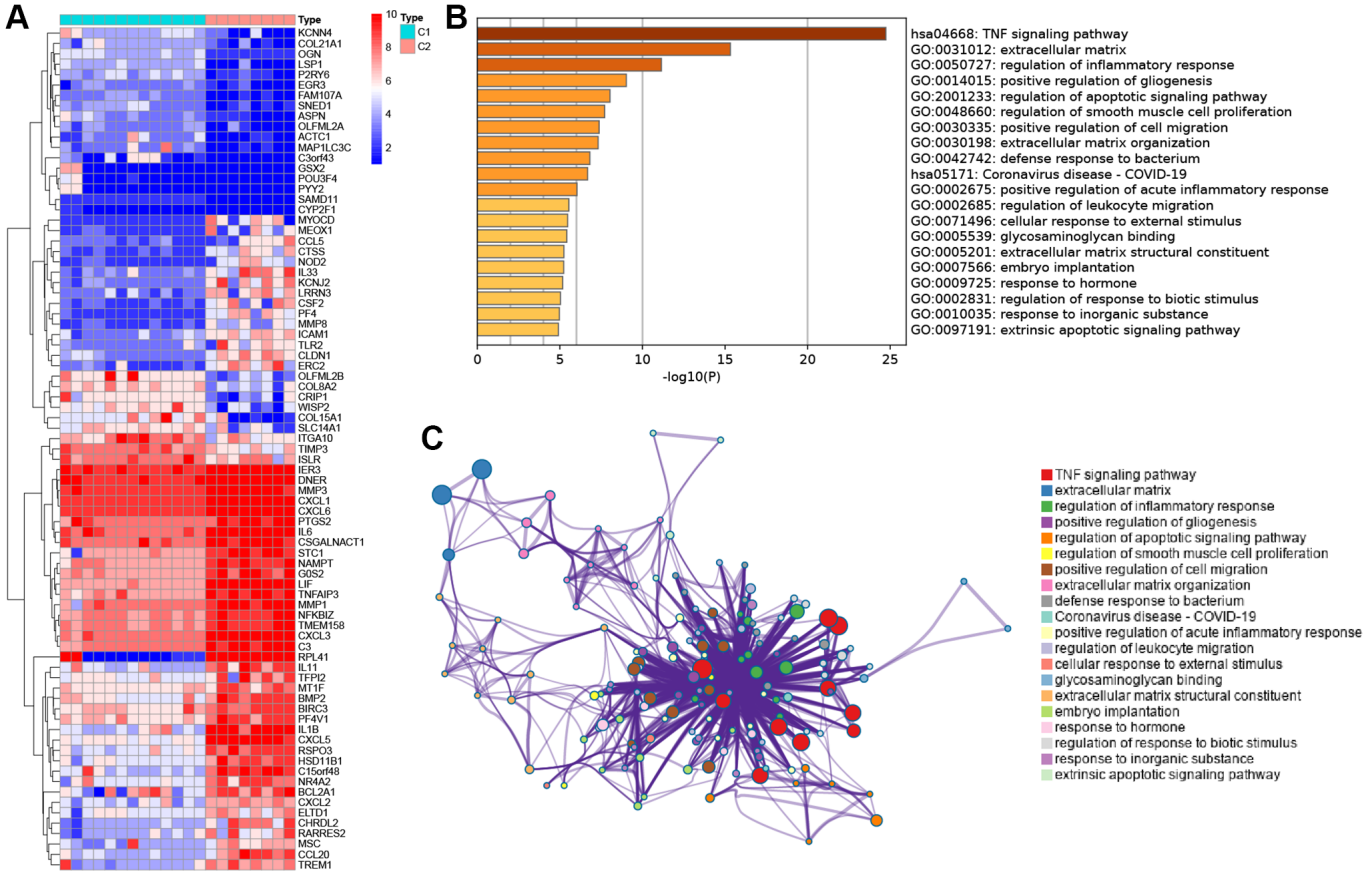
**Functional enrichment analysis between the two subtypes.** (**A**) Heatmap showing differentially expressed genes in the C1 and C2. (**B**) Functional enrichment of biological process and KEGG pathway. (**C**) Network diagram of gene functional enrichment analysis.

To understand the underlying mechanisms between subtypes, we performed differential analysis. There were 81 DEGs between the two subtypes (Figure 3A). The expression levels of inflammation-related genes, including interleukins and chemokine, were shown to be significantly increased in condition cluster C2. To reveal the possible biological functions and enrichment pathways of the clustered DEGs, KEGG pathway analysis and GO analysis were performed. It was discovered that they were mostly enriched at TNF signaling pathway (Figure 3B, 3C).

### Identification of module genes related to subtypes

**Figure 4:**
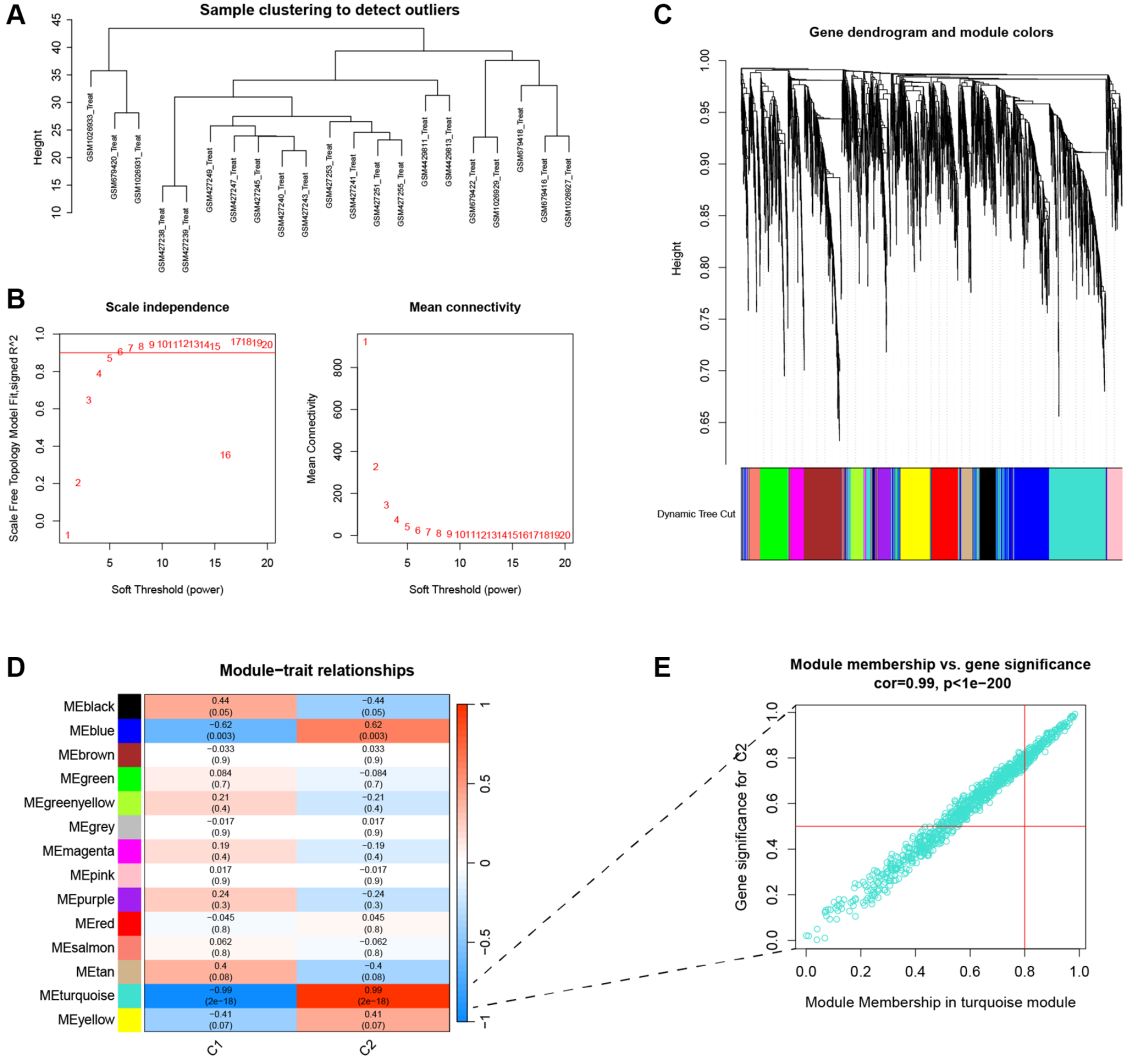
**Identification of key genes for clustering.** (**A**) Cluster dendrogram of 21 IVDD. (**B**) The scale independent value exceeded 0.9 with a low average connectivity when the soft threshold power was 6. (**C**) Cluster dendrogram of clustered genes to identify clinically meaningful modules associated with cluster occurrence. (**D**) Heatmap of the correlation between gene modules and clinical traits of IVDD. (**E**) The scatterplot of Gene Significance vs. Module Membership in the turquoise modules.

To obtain the key module genes of subtype, we performed WGCNA analysis on IVDD samples. Figure 4A shows a sample clustering map from the WGCNA algorithm that can be used to find possible biomarkers for AF type. Then, when the soft threshold power was 6, the scale-independent value exceeded 0.9 and the average connectivity was low (Figure 4B). The clustering tree of the degenerated disc-associated genes is shown in Figure 4C. The WGCNA network clustering analysis divided all genes into 14 modules (Figure 4D). The *P*-value of the turquoise module was less than 0.01, so this module was included in further analysis (Figure 4E).

### Identification of module genes related to disease

**Figure 5:**
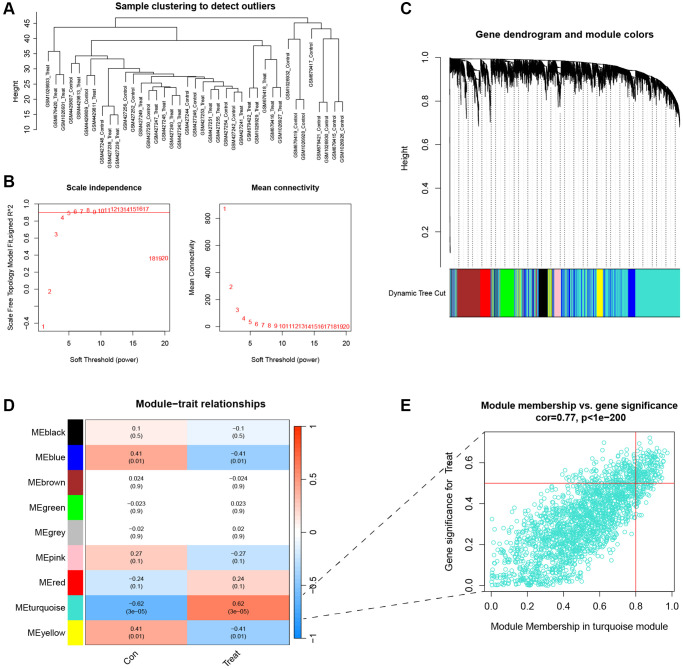
**Identification of key genes for IVDD.** (**A**) Cluster dendrogram of 39 IVD. (**B**) The scale independent value exceeded 0.9 with a low average connectivity when the soft threshold power was 5. (**C**) Cluster dendrogram of disease genes to identify clinically meaningful modules associated with IVDD development. (**D**) Heatmap of the correlation between gene modules and clinical traits of IVDD. (**E**) The scatterplot of Gene Significance vs. Module Membership in the turquoise modules.

Gene modules associated with IVDD were obtained by WGCNA analysis of the merged data, which included AF tissue from 18 degenerated discs and 21 normal discs. The clustering of the merged samples is shown in the Figure 5A. It was found that when the soft threshold power was 5, the scale-independent value exceeded 0.9 and the average connectivity was low (Figure 5B). A total of 10 modules were identified by averaging chain-level clustering (Figure 5C, 5D). Only the turquoise module in Figure 5D had a *P*-value less than 0.01, so this, comprising 156 genes, was included for further analysis (Figure 5E).

### Nomogram of subtype identification

**Figure 6:**
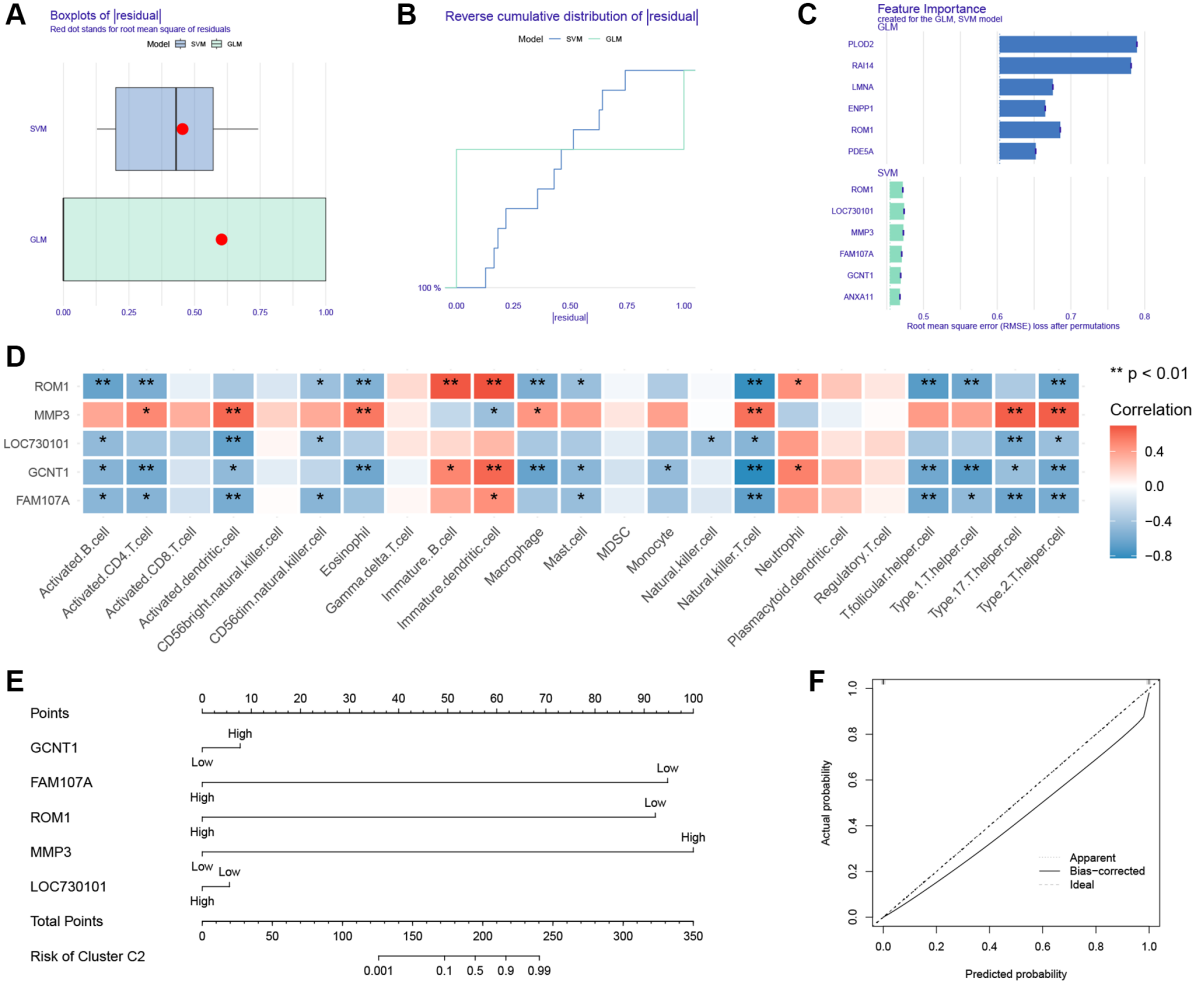
**Construction of nomogram.** (**A**) Boxplot of the residuals of the sample. (**B**) Cumulative residual distribution plot of sample. (**C**) The importance of variables in the GLM and SVM models. (**D**) Heatmap of correlation between ssGSEA algorithm results and hub genes. (**E**) Nomogram. (**F**) Calibration curve for the nomogram.

There were 154 genes in the clustering turquoise module and 156 genes in the IVDD turquoise module. The intersection of these two modules was then investigated, identifying a total of 80 key genes (Supplementary Figure 2). To further narrow the range of key module genes, GLM and SVM models were developed separately. Based on the cumulative residual distribution and box plots, SVM was found to be the best model due to the smallest sample residuals (Figure 6A, 6B). The first six explanatory variables of SVM were ROM1, LOC730101, MMP3, FAM107A, GCNT1, ANXA11, and the first six explanatory variables of GLM were ROM1, PLOD2, RAI14, LMNA, ENPP1, PDE5A (Figure 6C).

We used the SVM-identified genes as hub genes. The immunologic infiltration of IVDD has been determined by ssGSEA. A heatmap illustrating the hub genes that are closely linked with a majority of immune cells (Figure 6D). Previously we identified the presence of two subtypes of IVDD. Nomograms were developed for use in classifying subtypes (Figure 6E). The calibration curves demonstrate the nomogram’s high prediction accuracy (Figure 6F).

### ROM1 is lowly expressed in IVDD

**Figure 7:**
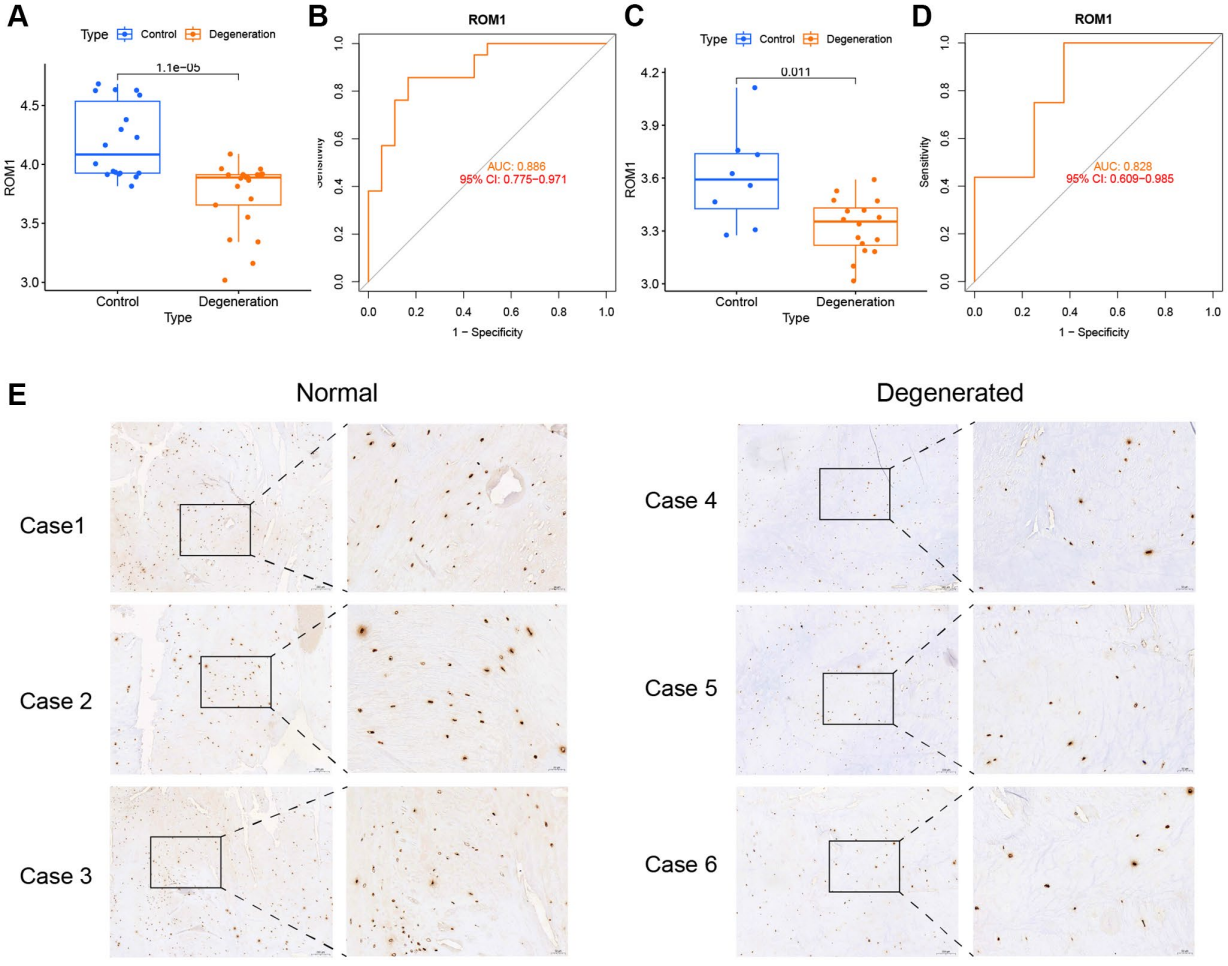
**The expression levels of ROM1.** (**A**) ROM1 expression was markedly lower based on the merged datasets (GSE41883, GSE27494, GSE17077, and GSE147383). (**B**) ROC analysis of ROM1 in merged datasets. (**C**) ROM1 expression in the external validation set (GSE70362). (**D**) ROC analysis of ROM1 in the external validation set. (**E**) Immunohistochemical staining showing low levels in IVDD.

ROM1 was the only gene present in both models, and we chose ROM1 for further analysis. The box plots showed low expression of ROM1 in degenerated AF (Figure 7A). To illustrate the ability of ROM1 as a diagnostic marker, we plotted a ROC curve for it, resulting in an AUC value of 0.886 (Figure 7B). Meanwhile, the GSE70362 dataset was used as a validation set for the external validation. ROM1 also showed low expression in the degenerated AF (*p* < 0.05) (Figure 7C) and the area under the ROC curve was observed to be 0.828 (Figure 7D). Clinical samples validated by immunohistochemistry also showed its low expression in IVDD (Figure 7E).

### The potential mechanisms for ROM1

**Figure 8:**
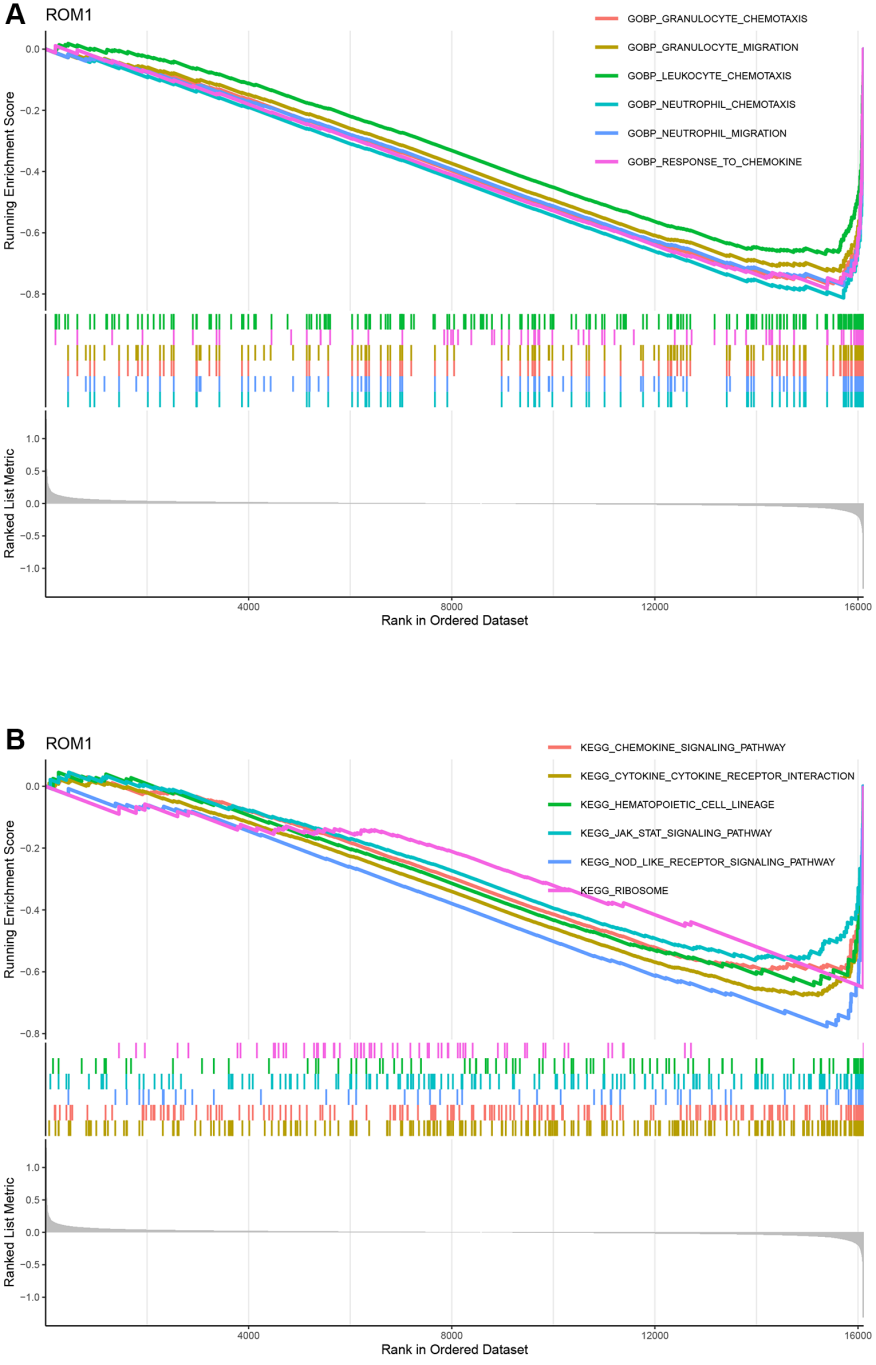
**The GO entries and KEGG pathways.** (**A**) GO terms. (**B**) KEGG pathways.

To explore potential mechanisms of ROM1, we then performed a GSEA for individual gene based on the GO and KEGG gene sets. In the GO analysis, ROM1 were involved in “neutrophil_chemotaxis”, “neutrophil_migration”, “granulocyte_chemotaxis”, “granulocyte_migration”, “response_to_chemokine”, and “leukocyte_chemotaxis” (Figure 8A). According to the KEGG pathway analysis, all diagnostic genes were found to participate in “cytokine_cytokine_receptor_interaction”, “chemokine_signaling_pathway”, “Nod-like_receptor_signaling_pathway”, “JAK/STAT_signaling_pathway” (Figure 8B). The above results illustrate that ROM1 is mainly involved in inflammation-related biological processes and pathways.

### The pan-cancer analysis of ROM1

**Figure 9:**
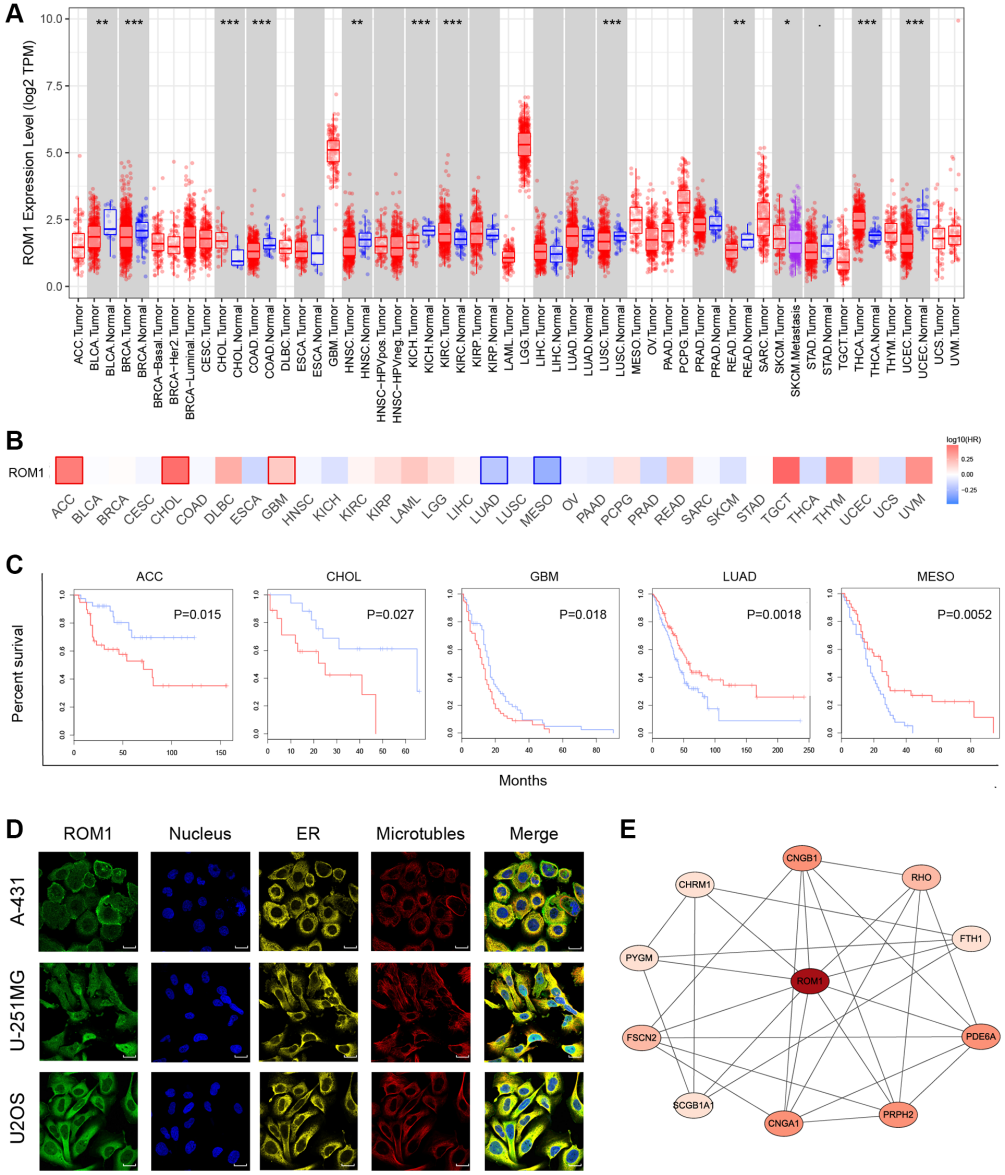
**Pan-cancer analysis of ROM1.** (**A**) Pan-cancer study of ROM1 expression based on the TIMER. (**B**) ROM1’s survival map in pan-cancer. (**C**) Kaplan-Meier survival curves for overall survival. (**D**) Immunofluorescence images of ROM1 protein expression in the nucleus, endoplasmic reticulum (ER), and microtubules in U2OS, U-251MG and A-431 cells (scar bar = 20 μm). (**E**) The PPI network of ROM1.

Further investigation has been conducted to examine the expression pattern of ROM1 over various types of cancer. The findings indicated that the expression of ROM1 was notably elevated in cancers associated with CHOL, KIRC, and THCA. On the other hand, a reduction in expression levels was seen in several tumor types, including BLCA, BRCA, and COAD et al. (Figure 9A). Furthermore, a prognostic analysis has been done to examine the influence of ROM1 on cancer prognosis (Figure 9B). The Kaplan-Meier analysis revealed that ROM1 has a potential association with increased risk for ACC, CHOL, and GBM, while displaying a potential protective effect for LUAD and MESO (Figure 9C). In addition, immunofluorescence (IF) staining showed the distribution and localization of ROM1 in different cells (Figure 9D). Finally, a network of protein-protein interaction (PPI) was constructed using the interaction data obtained from the STRING website (Figure 9E). The results mentioned above underscore the significant prognostic relevance of ROM1 in the context of pan-cancer.

## DISCUSSION

IVDD is an important contributor to LBP. However, IVDD is also present in more than 50% of patients without LBP [24, 25]. Thus, many of these individuals do not receive timely interventions for early prevention and treatment, which is one of the main reasons for the poor prognosis of patients. It is crucial to identify the various molecular subtypes that are linked to IVDD in order to advance our understanding of this condition. In recent years, data-mining strategies and comprehensive bioinformatics analyses using publicly accessible databases have proven to be effective methods for identifying potential biomarkers and even new therapeutic targets in complex diseases [25–27]. Therefore, the aim of this study was to explore different subtypes of IVDD and to identify relevant hub genes in AF cells.

In this study, we extracted AF transcriptomics data from multiple datasets through a series of bioinformatics analyses and screened genes with diagnostic value in subsequent analyses. First, we performed consensus clustering analysis based on DEFRGs to identify two subtypes and explore the underlying mechanisms associated with both subtypes. However, we did not perform a more in-depth analysis due to the lack of clinical data. Subsequently, we performed WGCNA analysis on the disease and subtype samples separately, which were processed by determining their intersection, leading to the identification of 80 genes. Then, with the hub genes identified by SVM, we were able to identify different subtype. Low expression of ROM1 was identified through external datasets and clinical samples. The single-gene GSEA analysis revealed the possible association with GO and KEGG. To understand more about ROM1, we performed a pan-cancer analysis of it and found that it affected the prognosis of multiple cancers.

Previously, most studies have focused on the NP tissue of the IVD. Zhang et al. identified inflammatory response-related features using WGCNA and LASSO, and explored the relationships between them and immune infiltration in IVDD. In addition, IL-1β, LYN, and NAMPT were validated as possible biomarkers [28]. Zhu et al. constructed good genetic and lncRNA diagnostic models by systematic analysis of lncRNA and microRNA expression profiles and lncRNA and miRNA expression between IVDD and healthy patients [29]. These studies provide new perspectives on the diagnosis and treatment of IVDD.

As an important component of the IVD, the AF has a role in maintaining the nutritional supply of the IVD [30]. At the same time, as a composite pressure vessel, it has the ability to generate hydrostatic pressure around the NP and resist loading [31, 32]. The structural stability of the disc tissue is important for the prevention of IVDD. Therefore, we explore the IVDD based on the perspective of the transcriptome of the AF. Some investigators have also used AF tissue to identify diagnostic markers for IVDD. Chen et al. used an integrated bioinformatics approach to generate a comprehensive overview of the gene networks associated with the AF in IVDD. This identified DSE, IL17RD, DUSP18, ROBO3, BANK1, MRC2, LGALSL, TFPI, GAP43, and HYAL1 as hub genes [33]. By constructing a PPI network, Li et al. found that the MMP2 and AGE-RAGE signaling pathways as well as the estrogen signaling pathway may play important roles in the onset and development of IVDD [34].

Research has demonstrated that cancer can be categorized into several immunological subtypes based on the extent of immune cell infiltration [35]. This classification can serve as a more accurate predictor of the tumor’s tumor microenvironment. Immune infiltration also has a significant impact on the progression of IVDD [36]. The extensive body of literature indicates that the infiltration of inflammatory elements following IVDD may contribute to discogenic pain [37]. However, the specific relationship between the extent of inflammatory infiltration and the degree of degeneration is still not well understood. There were two subtypes that we found, one of which was cluster C2 and had a significant level of immune infiltration. Additionally, a higher inflammatory activation status was discovered to be associated with the cluster C2 through the utilization of differential gene enrichment analysis between subtypes. The results demonstrated a similarity. Finally, hub genes were shown to have high relationships with immune cells.

ROM1 (retinal outer segment membrane protein 1) is a member of a photoreceptor-specific gene family and encodes an integral membrane protein found in the photoreceptor disk rim of the eye. This protein can either form homodimers or can heterodimerize with another photoreceptor, slowing retinal degeneration. It is essential for the morphogenesis of the disk rim, and may also function as an adhesion molecule involved in the stabilization and compaction of disks in the outer segment or the maintenance of the curvature of the rim. In recent years, several studies have shown that ROM1 is a key gene in a variety of diseases. Using various bioinformatics analysis tools, Wang et al. identified seven novel central genes (MME, ALB, CXCL12, CDH1, PROM1, ICAM1, and PTPRC) that may play key roles in the tumorigenesis of human clear cell renal cell carcinoma [38] and Wang et al. demonstrated that a set of five subtype-specific gene markers (CLUL1, CNGB1, ROM1, LRRC39, and RDH12) have the ability to predict retinoblastoma progression with excellent accuracy [39]. Ma et al. found that ROM1 regulates tumorigenesis and lung cancer progression for which it is a prognostic and therapeutic biomarker [40]. To our knowledge, the present study is the first to identify ROM1 as a diagnostic biomarker for IVDD using a publicly available GEO dataset and a comprehensive bioinformatics approach, which could provide new insights into the molecular mechanisms associated with the pathogenesis of IVDD.

The following limitations of our study need to be noted. Initially, we distinguished between two subtypes of patients with IVDD with different immune infiltrative status; however, due to the lack of additional clinical data pertaining to the patients, further investigation into the subtype differences was not feasible. Second, the sample size of the study was small, which may have led to some bias in the results. Finally, it is important to verify ROM1 expression levels at several levels.

## CONCLUSION

Overall, we identified two different subtypes of IVDD by various bioinformatics analyses. There are significant differences in the expression and enrichment pathways in different molecular subtypes of IVDD, which may be a key factor leading to heterogeneity in the clinical symptoms and prognosis of patients with IVDD. At the same time, we identified key genes for IVDD. Our current study provides novel diagnostic and therapeutic biomarkers for molecular subtypes of IVDD.

## Supplementary Materials

Supplementary Figures
