# Supplementary material for: Comprehensive analysis of gene expression profiles of annulus fibrosus subtypes and hub genes in intervertebral disc degeneration
Source: Aging (Albany NY). 2024 Mar 13;16(6):5370–86. doi: 10.18632/aging.205653 (PMC11006460; doi:10.18632/aging.205653)
Supplement: Supplementary Figures [file aging-16-205653-s001.pdf]

SUPPLEMENTARY FIGURES

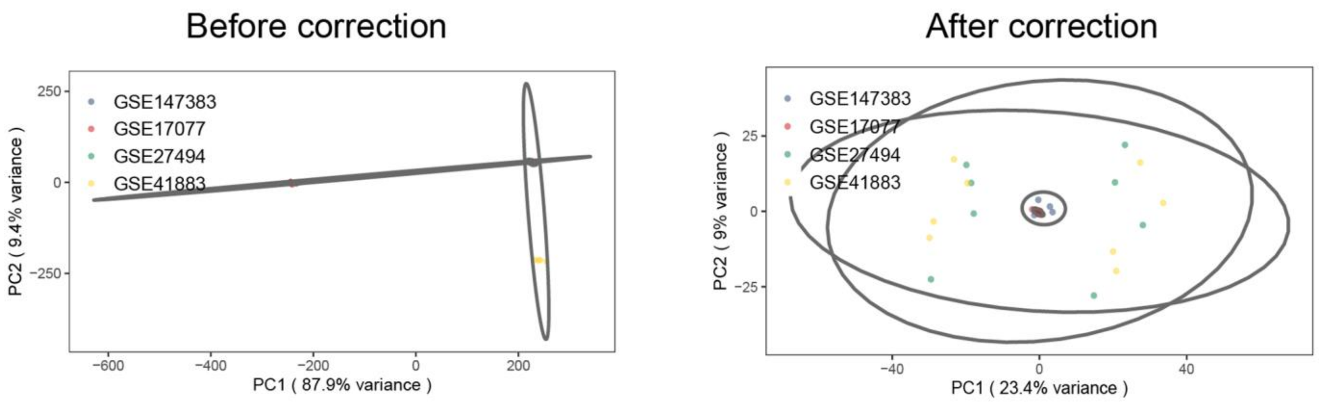

Supplementary Figure 1. PCA plots of different GEO datasets before and after removing the batch effect.

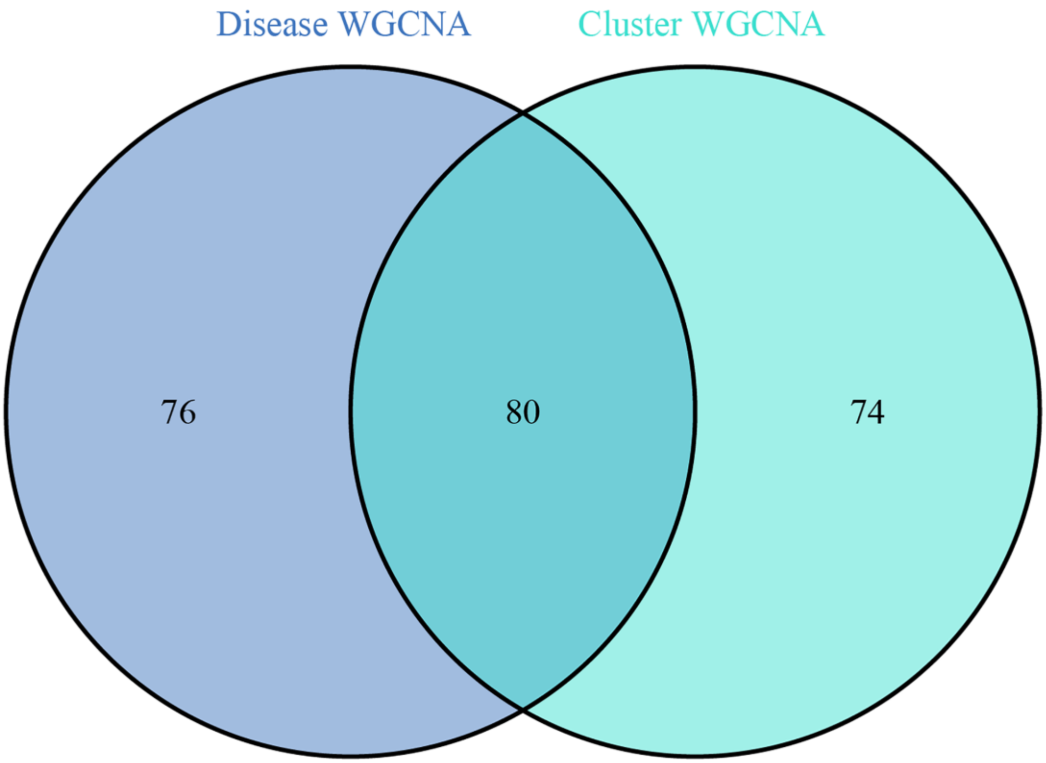

Supplementary Figure 2. Key genes by the intersection of the disease key module genes and the clustering key module genes.
